# Hypertrophic cardiomyopathy with midventricular obstruction and apical aneurysm formation in a single family: case report

**DOI:** 10.1186/1476-7120-7-26

**Published:** 2009-06-16

**Authors:** Georgios K Efthimiadis, Christodoulos Pliakos, Efstathios D Pagourelias, Despina G Parcharidou, Georgios Spanos, Stylianos Paraskevaidis, Ioannis H Styliadis, Georgios Parcharidis

**Affiliations:** 1 Cardiology Department, Medical School, Aristotle University of Thessaloniki, Thessaloniki, Greece; 2 Eurodiagnosi Medical Center, Thessaloniki, Greece

## Abstract

**Background:**

Hypertrophic cardiomyopathy (HCM) is an extremely heterogeneous disease. An under recognized and very often missed subgroup within this broad spectrum concerns patients with left ventricular (LV) apical aneurysms in the absence of coronary artery disease.

**Case presentation:**

We describe a case of HCM with midventricular obstruction and apical aneurysm formation in 3 patients coming from a single family. This HCM pattern was detected by 2D-echocardiography and confirmed by cardiac magnetic resonance imaging. A cardioverter defibrillator was implanted in one of the patients because of non-sustained ventricular tachycardia detected in 24-h Holter monitoring and an abrupt drop in systolic blood pressure during maximal exercise test. The defibrillator activated 8 months after implantation by suppression of a ventricular tachycardia providing anti-tachycardia pacing. The patient died due to refractory heart failure 2 years after initial evaluation. The rest of the patients are stable after a 2.5-y follow-up period.

**Conclusion:**

The detection of apical aneurysm by echocardiography in HCM patients may be complicated. Ventricular tachycardia arising from the scarred aneurysm wall may often occur predisposing to sudden death.

## Background

Hypertrophic cardiomyopathy (HCM) is an extremely heterogeneous disease, in terms of clinical course and phenotypic expression [1-3]. An under recognized and very often missed subgroup within this broad spectrum concerns patients with left ventricular (LV) apical aneurysms in the absence of coronary artery disease [4-10]. In this report we present a case-series of HCM patients with midventricular obstruction and apical aneurysm formation in a single family.

## Case presentation

**Figure 1:**
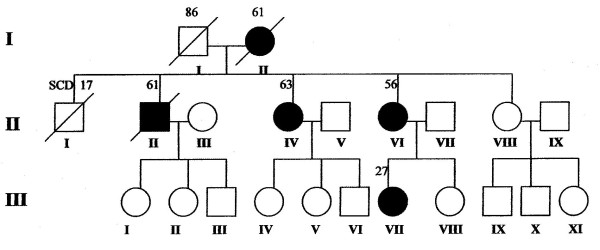
**Family tree**. Family tree showing hypertrophic cardiomyopathy (HCM) phenotypic expression through 3 generations. The three female patients we studied (case-index 1, 63-y; case-index 2, 56-y; case-index 3, 27-y) are represented by black colour filled circles in generations II and III. SCD: Sudden Cardiac death. Squares represent males and circles females. Black colour filled schemes represent HCM patients. Schemes erased by diagonal lines refer to deceased family members.

**Figure 2:**
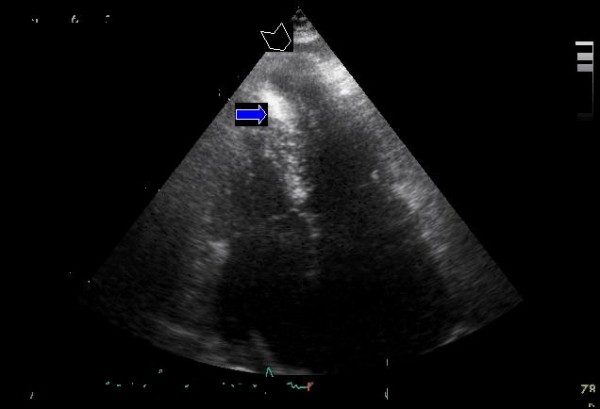
**Two dimensional transthoracic echocardiogram**. Apical four chamber view showing predominant thickening of midseptal and midlateral regions during systole (blue arrow) and the formation of left ventriculat apical aneurysm (arrow head) in case index-1 patient.

**Figure 3:**
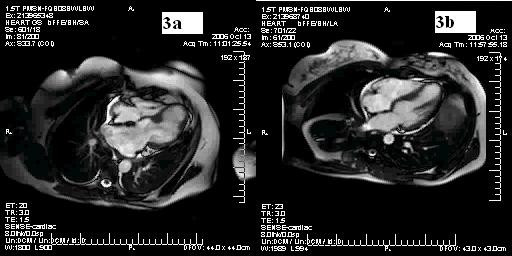
**Cardiac Magnetic Resonance Images**. 3a) Cardiac magnetic resonance image (MRI) showing midventricular systolic thickening and 'hourglass' left ventricular shape in case index-2 patient and her daughter case-index 3(3b). Note the formation of apical aneurysms in both cases.

**Figure 4:**
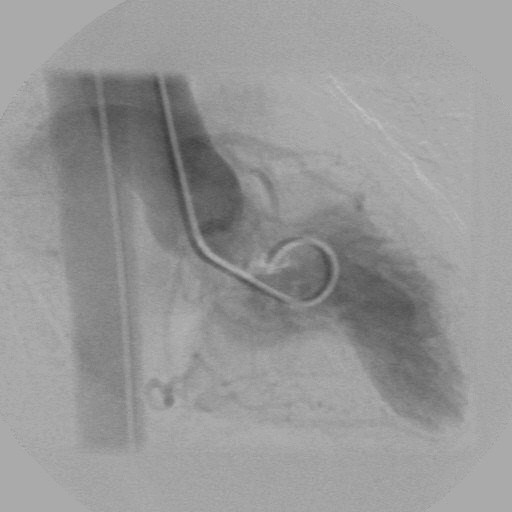
**Cardiac catheterization image**. Image obtained during catheterization of case index-2 patient showing systolic thickening of midventricular septum along with apical aneurysm formation.

We studied 3 female patients (case-index 1, 63-y; case-index 2, 56-y; case-index 3, 27-y) (Figure 1) with already diagnosed HCM who were referred to our department for further evaluation. NYHA class was graded as III in the case-index 1 and as II in the case-index 2 and 3 patients. The patients underwent electrocardiography, echocardiography, 24-h Holter monitoring, treadmill cardiopulmonary exercise test, and cardiac magnetic resonance imaging. Unfortunatelly, genotype analysis was not performed since it is not, at present, available in our laboratory. Coronary arteriography performed in case-index 1 and case-index 2 patients excluded significant atherosclerotic narrowing of the extramural coronary arteries. The distribution of LV hypertrophy assessed by 2-D echo was similar among patients, with predominant thickening in the midseptal and midlateral regions (maximum wall thickness 16–18 mm) (Figure 2, Additional File 1). None of the patients showed LV outflow tract obstruction. A formation of a wall-thinning aneurysm involving the LV apex was detected in all 3 patients, a finding also confirmed by means of cardiac magnetic resonance imaging (MRI) and by cardiac catheterization (Figures 3, 4 and Additionals file 2, 3). A cardioverter defibrillator was implanted in index-case 1 patient because of non-sustained ventricular tachycardia detected in 24-h Holter monitoring and an abrupt drop in systolic blood pressure during maximal exercise test. The defibrillator activated 8 months after implantation by suppression of a ventricular tachycardia providing anti-tachycardia pacing. The patient died due to refractory heart failure 2 years after initial evaluation. The rest of the patients are stable after a 2.5-y follow-up period.

## Discussion

In this report we present a case of HCM with midventricular obstruction and apical aneurysm formation in 3 patients coming from a single family.

Isolated midventricular obstruction is an uncommon phenotype of HCM [7,11]. The midcavity obstruction is due to the midsystolic muscular apposition of the septum and LV free wall producing distinct proximal and distal chambers, resembling an "hourglass" shape of LV. Maron MS et al reported that the formation of LV apex aneurysm in HCM accounts approximately for 2% of HCM population. The pattern of hypertrophy in such patients is that of midventricular obstruction in 68%, and that of apical hypertrophy in 32% of patients [5]. Unfortunatelly, genotype analysis was not performed in our patients since it is not available in our laboratory. However, this disorder has been associated with mutations of the essential or regulatory light chains of myosin. These types of mutation are assumed to cause stretch activation response disrupt leading to midventricular obstruction [12].

The detection of apical aneurysms by echocardiography in HCM patients may be complex, if hypertrophy is confined to posterior septum, LV free wall, or to the apex. Furthermore, echocardiographist's experience, the quality of acoustic window and acquired images along with aneurysm's size play an important role in the aneurysm identification. According to a previous study, echocardiography identified only 57% of HCM patients having aneurysms, all of which were either medium or large [5]. On the contrary cardiac MRI, showing a greater spatial resolution, proves to be 100% sensitive in revealing this entity [5,13].

The mechanism responsible for the formation of apical aneurysms in HCM patients remains unresolved while several causes have been accused, such as increased LV wall stress as a result of midcavitary LV obstruction and elevated intracavitary systolic pressures, genetic predisposition and myocardial bridging of the left anterior descending coronary artery [5].

The formation of apical aneurysm in HCM represents a very serious clinical form of the disease. Very often, LV systolic function is depressed and thrombi may be developed inside the aneurysm leading to embolic events. Ventricular tachycardia arising from the scarred apical wall may also occur, predisposing to sudden death.

## Conclusion

LV apical aneurysms affect up to 2% of the entire HCM population. Their detection using 2D echocardiography may be difficult. MRI imaging, on the contrary, shows 100% sensitivity in revealing them. The presentation of apical aneurysms in HCM patients is connected to serious complications including embolic events or even sudden death.

## Consent

Written informed consent was obtained at first visit from all patients for publication of this case report and all accompanying images. A copy of the written consent is available for review by the Editor-in-Chief of this journal.

## Competing interests

The authors declare that they have no competing interests.

## Authors' contributions

GKE and EP conceived the case report, collected the data, reviewed literature and wrote the manuscript. IHS and GP revised the article for important intellectual content and edited the final version. GKE, DGP and CP performed the ultrasounds and participated in the analysis and interpretation of data. GS performed cardiac MRI. SP implanted the ICD. All authors read and approved the final manuscript.

## Supplementary Material

Additional file 1 **Transthoracic echocardiography movie**. This movies shows an apical two chamber view during 3 cardiac circles demonstrating mid-ventricular septal and lateral walls thickening and apical aneurysm formation in case-index 1 patient. Click here for file

Additional file 2 **Cardiac Magnetic Resonance movie**. Cardiac MRI movie obtained from case index-2 patient showing systolic midventricular wall thickening and an apical aneurysm. Click here for file

Additional file 3 **Catheterization movie**. Catheterization movie revealing systolic left midventricular obstruction and apical aneurysm in case-index 2 patient. Click here for file

## References

[B1] Maron BJ (2002). Hypertrophic cardiomyopathy: a systematic review. JAMA.

[B2] Wigle ED, Rakowski H, Kimball BP, Williams WG (1995). Hypertrophic cardiomyopathy: clinical spectrum and treatment. Circulation.

[B3] Klues HG, Schiffers A, Maron BJ (1995). Phenotypic spectrum and patterns of left ventricular hypertrophy in hypertrophic cardiomyopathy: morphologic observations and significance as assessed by two-dimensional echocardiography in 600 patients. J Am Coll Cardiol.

[B4] Alfonso F, Frenneaux MP, McKenna WJ (1989). Clinical sustained uniform ventricular tachycardia in hypertrophic cardiomyopathy: association with left ventricular apical aneurysm. Br Heart J.

[B5] Maron MS, Finley JJ, Bos JM, Hauser TH, Manning WJ, Haas TS, Lesser JR, Udelson JE, Ackerman MJ, Maron BJ (2008). Prevalence, clinical significance, and natural history of left ventricular apical aneurysms in hypertrophic cardiomyopathy. Circulation.

[B6] Zenovich AG, Lesser JR, Hanna CA, Maron BJ (2006). Identical twins with hypertrophic cardiomyopathy and apical aneurysm. Am J Cardiol.

[B7] Fighali S, Krajcer Z, Edelman S, Leachman RD (1987). Progression of hypertrophic cardiomyopathy into a hypokinetic left ventricle: higher incidence in patients with midventricular obstruction. J Am Coll Cardiol.

[B8] Matsubara K, Nakamura T, Kuribayashi T, Azuma A, Nakagawa M (2003). Sustained cavity obliteration and apical aneurysm formation in apical hypertrophic cardiomyopathy. J Am Coll Cardiol.

[B9] Nakamura T, Matsubara K, Furukawa K, Azuma A, Sugihara H, Katsume H, Nakagawa M (1992). Diastolic paradoxic jet flow in patients with hypertrophic cardiomyopathy: evidence of concealed apical asynergy with cavity obliteration. J Am Coll Cardiol.

[B10] Wigle ED, Rakowski H (1992). Hypertrophic cardiomyopathy: when do you diagnose midventricular obstruction versus apical cavity obliteration with a small nonobliterated area at the apex of the left ventricle?. J Am Coll Cardiol.

[B11] Falicov RE, Resnekov L (1977). Midventricular obstruction in hypertrophic obstructive cardiomyopathy. New diagnostic and therapeutic challenge. Br Heart J.

[B12] Poetter K, Jiang H, Hassanzadeh S, Master SR, Chang A, Dalakas MC, Rayment I, Sellers JR, Fananapazir L, Epstein ND (1996). Mutations in either the essential or regulatory light chains of myosin are associated with a rare myopathy in human heart and skeletal muscle. Nat Genet.

[B13] Moon JC, Fisher NG, McKenna WJ, Pennell DJ (2004). Detection of apical hypertrophic cardiomyopathy by cardiovascular magnetic resonance in patients with non-diagnostic echocardiography. Heart.

